# Diagnostic Value of Muscle [^11^C] PIB-PET in Inclusion Body Myositis

**DOI:** 10.3389/fneur.2019.01386

**Published:** 2020-01-17

**Authors:** Yu-ichi Noto, Masaki Kondo, Yukiko Tsuji, Shigenori Matsushima, Toshiki Mizuno, Takahiko Tokuda, Masanori Nakagawa

**Affiliations:** ^1^Department of Neurology, Kyoto Prefectural University of Medicine, Kyoto, Japan; ^2^Department of Radiology, Kyoto Prefectural University of Medicine, Kyoto, Japan; ^3^Department of Molecular Pathobiology of Brain Diseases, Kyoto Prefectural University of Medicine, Kyoto, Japan; ^4^North Medical Center, Kyoto Prefectural University of Medicine, Kyoto, Japan

**Keywords:** inclusion body myositis, idiopathic inflammatory myopathy, dermatomyositis, polymyositis, amyloid, PIB-PET, diagnosis

## Abstract

**Background:** The accumulation of multiple-protein aggregates within muscle fibers is a pathological hallmark of sporadic inclusion body myositis (s-IBM) with the presence of inclusion bodies. Amyloid-beta is one of the accumulated proteins in s-IBM. The aim of this study was to elucidate the utility of Pittsburgh compound B-positron emission tomography (PIB-PET) for diagnosing s-IBM.

**Methods:** Nine patients with s-IBM and four patients with idiopathic inflammatory myopathy (IIM) were included. Patients underwent PIB-PET of body muscles. Standardized uptake values (SUVs) were measured in 16 muscles. A comparison of SUVs was made between s-IBM and IIM groups. The correlation between PIB-PET and clinical parameters was analyzed.

**Results:** The mean SUV of all muscles in s-IBM patients was higher than in IIM patients (0.32 vs. 0.25, respectively; *p* = 0.031). Subgroup analysis identified a clear difference in SUVs of the forearm and lower-leg muscle groups (*p* = 0.021 and *p* = 0.045, respectively). There was no correlation between SUVs and clinical parameters in s-IBM patients.

**Conclusions:** Muscle PIB-PET may help to make a diagnosis of s-IBM.

## Introduction

Sporadic inclusion body myositis (s-IBM) is the most frequent progressive muscle disease associated with aging. The diagnostic approach using needle electromyography is sometimes challenging because the amplitude of motor units can be large in s-IBM. The accumulation of misfolded, ubiquitinated, congophilic, and multiple-protein aggregates within muscle fibers is a pathological hallmark of s-IBM with the presence of inclusion bodies (1). Amyloid-beta, which is the main component of amyloid plaques found in the brains of patients with Alzheimer's disease (AD), is one of the accumulated proteins in s-IBM. To visualize the pathological protein in the brain, Pittsburgh compound B-positron emission tomography (PIB-PET) imaging is used as a diagnostic tool for AD (2). A preliminary study using PIB-PET to detect amyloid-beta deposition in limb muscles has been performed in a s-IBM cohort and found increased PIB uptake in the lower limb muscles in some s-IBM patients. However, it still remains to be elucidated whether this will be reliable enough to be of diagnostic value (3). The aim of this study was to elucidate the utility of trunk and limb muscle PIB-PET for the diagnosis of s-IBM.

## Methods

### Subjects

Nine patients with s-IBM were included. As disease control subjects, four patients with idiopathic inflammatory myopathy (IIM) were also included. The diagnosis was based on the European Neuromuscular Center (ENMC) IBM research diagnostic criteria 2011 for s-IBM patients and by the 2004 ENMC criteria for IIM patients (except s-IBM) (4, 5). Demographic and clinical details including the total Medical Research Council (MRC) scale sum score were collected by experienced neurologists. Additionally, the IBM functional rating scale (IBMFRS) was applied to s-IBM patients (6). All participants gave written informed consent, approved by the ethics committee of Kyoto Prefectural University of Medicine.

### Positron Emission Tomography

Forty-five minutes after slow bolus intravenous injection of ^11^C-labeled PIB ([^11^C]PIB), whole-body PET scanning was performed with a Siemens ECAT ACCEL scanner (Siemens Medical Systems, Erlangen, Germany) equipped with LSO crystals (3D mode, 47 planes, 16.2-cm axial field of view). Subjects were measured from the lower leg to neck according to a previous study (3). The PET acquisition time was 3 min per single bed. In our PET scan protocol, scanning is performed at 11 bed positions and it took 33 min to complete the scanning. Whole-body CT was also conducted to determine retention sites. Sixteen regions of interest (ROIs) were manually generated in truncal and limb muscles using axial images. Manual drawing of ROIs was conducted twice for each muscle by one examiner. Two experienced neurologists (YN and MK) who were familiar with muscle anatomy and blinded to the diagnosis of each patient were employed as examiners in this study to confirm inter-rater reliability. The 16 ROIs were: biceps brachii, triceps brachii, paraspinal (Th 10–12 level), quadriceps, hamstrings, tibialis anterior, gastrocnemius, and forearm muscle complexes on both sides. Examiners were instructed to designate an ROI in an axial image showing the largest muscle cross-sectional area in each muscle group based on visual assessment [Representative ROIs are shown in Supplementary File (S1)]. The mean muscle [^11^C]PIB retention was measured in each ROI using PMOD software (version 3.2, PMOD Technologies Ltd., Zurich, Switzerland). Mean retention was expressed in standardized uptake values [SUVs, local radioactivity retention divided by administered radioactivity per body mass (g/mL)].

### Statistics

Intra- and inter-rater reliabilities of [^11^C]PIB retention measurements were ascertained by calculating the intraclass correlation coefficient (ICC). Fisher's exact test was used to analyze the sex ratio in s-IBM and IIM patients. Biometric parameters and SUVs were compared between s-IBM and IIM patients using the independent *t*-test for parametric and Mann-Whitney U test for non-parametric data. Comparisons of SUVs were performed in all muscles, and in upper-arm (biceps brachii and triceps brachii), forearm (forearm muscle complexes), trunk (paraspinal muscle), thigh (quadriceps, hamstrings), and lower-leg (tibialis anterior, gastrocnemius) muscle groups using pooled data for each disease group. Mean SUV of four measurements by two examiners (YN and MK) was used for analysis as a SUV in each ROI. In SUVs of any muscle groups with a significant difference between s-IBM and IIM patients, receiver operating characteristic (ROC) curves were plotted to determine the diagnostic utility of [^11^C]PIB retention in the trunk and limb muscle PIB-PET. Appropriate cutoff values were determined using the Youden index. In the s-IBM group, the correlation between the SUVs and clinical variables (disease duration, MRC score, and IBMFRS) was assessed using a Pearson or Spearman correlation, as appropriate, with Bonferroni correction applied for multiple-correlation analyses. In all comparisons, *P* < 0.05 was considered significant. All statistical analyses were performed using JMP software 12.2 (SAS Institute, Cary, North Carolina, USA).

## Results

All patients with s-IBM fulfilled the criteria of clinically defined IBM in the ENMC IBM research diagnostic criteria 2011 (4). Patients with IIM consisted of three definite dermatomyositis and one non-specific myositis based on the 2004 ENMC criteria (5).

Regarding demographic and clinical parameters, no significant differences were evident in terms of the sex ratio, age, disease duration or the total MRC scale sum score between patients with s-IBM and IIM (Table 1). IBMFRS was 25.1 ± 10.7 (mean ± SD) in s-IBM patients.

**Table 1:** Demographic and clinical data.

	**IBM**	**IIM**	***p*-value**
	**(*n* = 9)**	**(*n* = 4)**	
Gender (M:F)	5:4	1:3	0.559
Age	73.8 (3.9)	68.0 (10.4)	0.486
Disease duration (months)	57.3 (37.0)	98.0 (151.1)	0.534
MRC scale sum score	45.8 (6.7)	51.8 (8.4)	0.174
IBMFRS	25.1 (10.7)	-	-

*Data are shown as mean (SD). IBM, inclusion body myositis; IIM, idiopathic inflammatory myopathy; MRC, Medical Research Council; IBMFRS, IBM functional rating scale*.

### Positron Emission Tomography Findings

**Figure 1:**
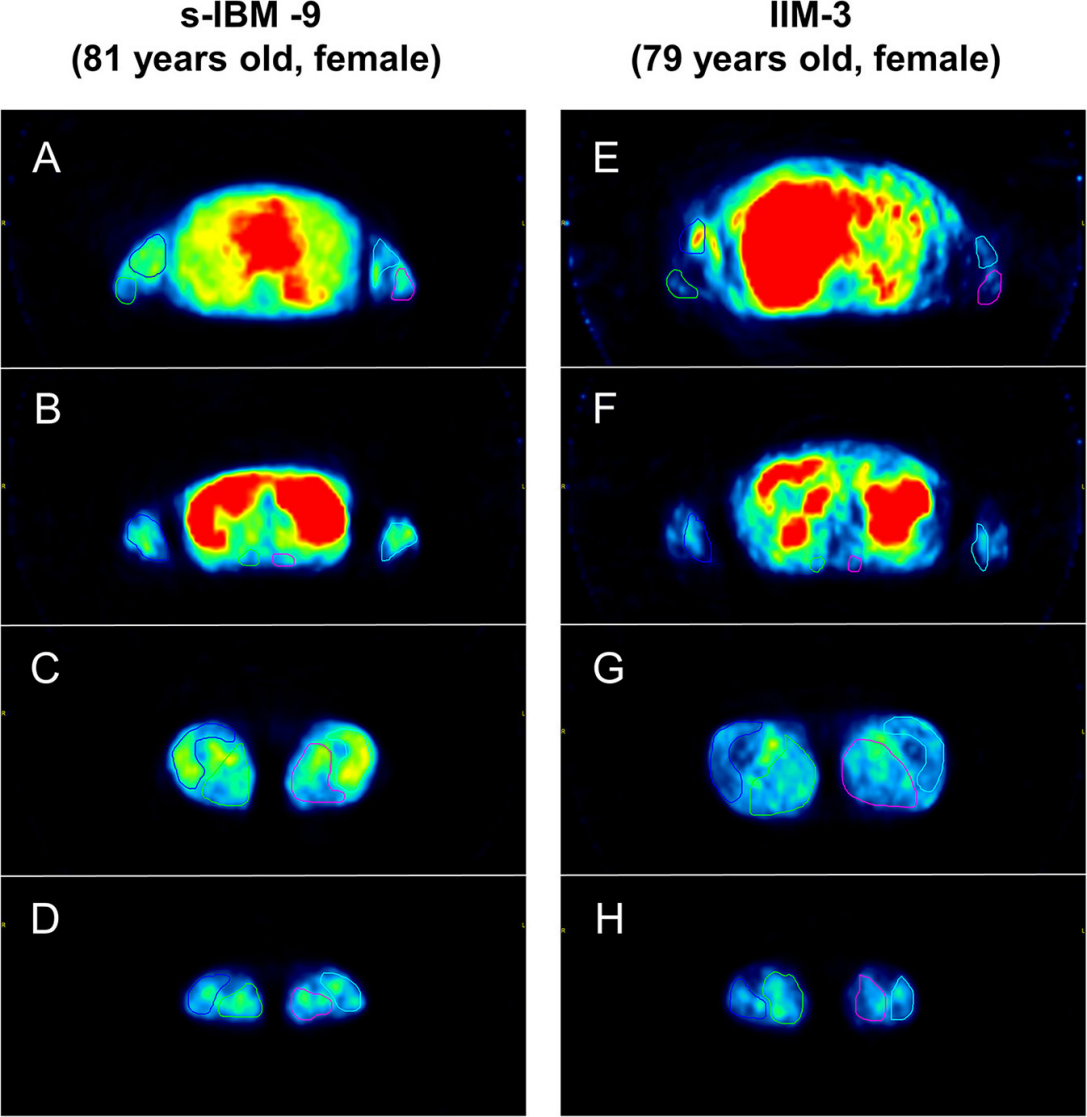
Representative images of Pittsburgh Compound B (PIB) uptake in limb and trunk muscles. Patient sporadic-inclusion body myositis (s-IBM)-9 had higher mean [^11^C]PIB-standardized uptake values (SUVs) in each muscle group than patient idiopathic inflammatory myopathy (IIM)-3 (dermatomyositis). [Upper arm muscle group: 0.30 vs. 0.18 **(A,E)**, forearm muscle group: 0.31 vs. 0.21 **(B,F)**, trunk muscle group: 0.32 vs. 0.24 **(B,F)**, thigh muscle group: 0.40 vs. 0.26 **(C,G)**, lower leg muscle group: 0.30 vs. 0.24 **(D,H)**].

The amount of bolus injection of [^11^C]PIB was 604.5 ± 70.1 MBq (mean ± SD) per subject. Reliability assessment of [^11^C]PIB retention measurements identified excellent intra-rater reliability with ICC of 0.96 (YN) and 0.93 (MK) and excellent inter-rater reliability with ICC of 0.85. Representative images of PIB uptake in s-IBM and IIM patients are presented in Figure 1. Pooled analysis of SUVs from 16 muscle groups in each patient demonstrated that the SUVs were significantly higher in the s-IBM than IIM group (Table 2). Analysis of each muscle group identified significant differences in the forearm and lower-leg muscle groups (Table 2).

**Table 2:** The comparison of the standardized uptake values.

	**IBM**	**IIM**	***p*-value**
All muscles	0.32 (0.06)	0.25 (0.01)	0.031^*^
Upper arm muscle group	0.31 (0.09)	0.23 (0.05)	0.123
Forearm muscle group	0.30 (0.07)	0.21 (0.02)	0.021^*^
Trunk muscle group	0.36 (0.09)	0.30 (0.10)	0.123
Thigh muscle group	0.32 (0.09)	0.26 (0.04)	0.217
Lower leg muscle group	0.32 (0.06)	0.25 (0.01)	0.045^*^

*Data are shown as mean (SD). IBM, inclusion body myositis; IIM, idiopathic inflammatory myopathy*.

* *< 0.05*.

### Receiver Operating Characteristic Curve Analysis

Using averaged SUVs of all muscle, forearm, and lower-leg muscle groups in each subject, ROC curve analysis was performed. To distinguish s-IBM from IIM, the optimal cutoff value was determined as 0.301 for the mean SUV of all muscles (i.e., 16 ROIs) (sensitivity: 88.9%, specificity: 100%), with the mean SUV of the forearm muscle of 0.249 (sensitivity: 88.9%, specificity: 100%), and the mean SUV of the lower-leg muscle group of 0.3 (sensitivity: 77.8%, specificity: 100%). AUC values were 0.89, 0.92, and 0.86, respectively.

### Correlation Between [^11^C]PIB Retention and Clinical Variables

In patients with s-IBM, there were no significant correlations between the mean SUV of all muscles and clinical variables (disease duration, MRC score, and IBMFRS).

## Discussion

The present study demonstrated that the level of [^11^C]PIB retention was significantly higher in muscles, especially forearm and lower-leg muscles, of s-IBM patients than in those of IIM patients. ROC analysis revealed that measurement of [^11^C]PIB retention is of diagnostic value in differentiating s-IBM from IIM. However, there was no correlation between [^11^C]PIB retention and clinical variables in s-IBM.

Maetzler et al. initially elucidated the possibility that [^11^C]PIB-PET detected muscular amyloid-beta in s-IBM patients *in vivo* (3). In that study, they measured [^11^C]PIB-SUV in four muscles (deltoid, finger flexors, vastus lateralis, and gastrocnemius muscles) and found a significant increase of the median [^11^C]PIB-SUV only in the gastrocnemius muscle of s-IBM patients compared with those of non-IBM patients. The present study revealed that SUVs in all muscles were higher in s-IBM than IIM patients. In addition, a clear increase in SUVs of the forearm and lower-leg muscles of s-IBM patients was evident. Unlike in the previous study, we measured SUVs of many muscles using axial images and conducted manual ROI tracing for the measurement of SUVs. Also, only IIM patients were included as disease control subjects in the present study. These differences in methodology may account for the discrepancy in results between the present and the previous study. Similar to the previous study by Maetzler et al., SUVs are not zero even in our IIM patients (3). They noted that increased PIB binding was detected mainly in medium- and large-size blood vessels in their non-IBM patient. ROIs generated in the present study also include blood vessels in the muscles. The existence of PIB in blood vessels may be one of the reasons why SUVs are not zero even in non-s-IBM patients. Also, we speculate that aging may cause the deposition of amyloid in muscles. To validate these hypotheses, muscle PIB-PET should be conducted in healthy subjects in the future study.

The quadriceps and forearm muscles are mainly affected in typical s-IBM patients (7). However, there was no significant difference in SUVs of the thigh muscles between the two groups in the present study, consistent with the results of the previous study. In addition, no correlation between the [^11^C]PIB retention level and clinical parameters was noted. A study of AD reported that the [^11^C]PIB retention level was not associated with the disease severity in advanced stages of the disease (8). The retention level of [^11^C]PIB may also not be associated with the severity of the disease in s-IBM as it shows the same pathology as AD. Furthermore, a difference in the [^11^C]PIB retention level was noted in forearm and lower-leg muscles. In IBM, amyloid-beta shows a tendency to accumulate in distal muscles in the present study. In support of this finding, Haczkiewicz et al. reported that abnormal pathological findings compatible with s-IBM were detected even in the gastrocnemius muscle of their s-IBM patient, although the presence of amyloid deposition was unclear in the muscle (9).

As study limitations, the number of patients was limited, and the patient population included those who had a long disease duration and/or were treated with long-term immunomodulatory therapy. To validate the utility of this novel method, further studies with a larger number of patients who are treatment-naïve and have a short disease duration are needed. Furthermore, patients with amyotrophic lateral sclerosis (ALS) or limb-girdle muscular dystrophy should be included as disease control subjects in future studies because s-IBM sometimes mimics ALS or LGMD in clinical and needle electromyography settings.

Regarding the diagnostic value of muscle PIB-PET, specificities of mean SUVs in all, forearm, and lower-leg muscles according to cutoff values determined by the Youden index were 100%, respectively, facilitating differentiation between s-IBM and IIM. Inter-rater and intra-rater reliabilities were also excellent on measuring [^11^C]PIB retention. In cases whereby muscle biopsy is difficult or sampling error occurs, muscle PIB-PET may help to make a diagnosis of s-IBM.

## Data Availability Statement

All datasets generated for this study are included in the article/Supplementary Material.

## Ethics Statement

The authors confirm that they have read the journal's position on issues involved in ethical publication and affirm that this report is consistent with those guidelines. The patient was evaluated at Kyoto Prefectural University of Medicine hospital under a protocol approved by the local ethics committee of Kyoto Prefectural University of Medicine. Written informed consent was obtained from the patient.

## Author Contributions

YN and MK: study design, acquisition of data, analysis, and interpretation of data, drafting and revising the manuscript for content. YT: acquisition of data, analysis, and interpretation of data. SM and TT: interpretation of data, drafting and revising the manuscript for content. TM and MN: study supervision and drafting and revising the manuscript for content.

### Conflict of Interest

The authors declare that the research was conducted in the absence of any commercial or financial relationships that could be construed as a potential conflict of interest.

## Abbreviations

AD: Alzheimer's disease
ALS: amyotrophic lateral sclerosis
ENMC: European Neuromuscular Center
IBMFRS: inclusion body myositis functional rating scale
ICC: intraclass correlation coefficient
IIM: idiopathic inflammatory myopathy
MRC: Medical Research Council
PET: positron emission tomography
PIB: Pittsburgh compound B
ROC: receiver operating characteristic
ROI: region of interest
s-IBM: sporadic inclusion body myositis
SUV: standardized uptake value.
